# Potent block of potassium channels by MEK inhibitor U0126 in primary cultures and brain slices

**DOI:** 10.1038/s41598-018-27235-1

**Published:** 2018-06-11

**Authors:** Jin-Zhao Wang, Cheng Long, Kai-Yuan Li, Hua-Tai Xu, Li-Lian Yuan, Gang-Yi Wu

**Affiliations:** 1 School of Life Sciences, South China Normal University, Guangzhou, 510631 China; 2 Institute of Neuroscience, Chinese Academy of Sciences, 320 Yue Yang Road, Shanghai, 200031 China; 3 Department of Physiology and Pharmacology, Des Moines University, Des Moines, IA 50312 USA

## Abstract

U0126 (1,4-diamino-2,3-dicyano-1,4-bis (2-aminophenylthio) butadiene), a widely used mitogen-activated protein kinase kinase (MEK) inhibitor, was found to accelerate voltage-gated K^+^ channel (K_V_) inactivation in heterologous cells expressing several types of K_V_. The goal of this study was to examine whether U0126 at a concentration thought to specifically inhibit MEK signaling also inhibits K_V_ in native neurons of primary cultures or brain slices. U0126 caused a dose-dependent inhibition of both the transient (I_A_) and sustained (I_DR_) components of K^+^ currents in hippocampal neurons. U0126 also exhibited much higher potency on the I_A_ and I_DR_ than the classical K_V_ blockers 4-aminopyridine (4-AP) and tetraethylammonium (TEA). Consistent with its inhibitory effect on K_V_, U0126 broadened action potential duration, profoundly affected the repolarizing phase, and dramatically reduced firing frequency in response to current pulse injections. Despite the potent and reversible action of U0126 on K_v_ channels, PD98059, a structurally-unrelated MEK inhibitor, did not induce such an effect, suggesting U0126 may act independently of MEK inhibition. Together, these results raise cautions for using U0126 as a specific inhibitor for studying MEK signaling in neurons; on the other hand, further studies on the blocking mechanisms of U0126 as a potent inhibitor of K_V_ may provide useful insights into the structure-function relationship of K_V_ in general.

## Introduction

The mitogen-activated protein kinase (MAPK), also known as extracellular signal-regulated kinase (ERK1/2) is activated by the dual phosphorylation catalyzed by MAPK kinase (MAPKK, also known as MEK). The MAPK cascade, one of the major intracellular signaling pathways, plays a key role in proliferation, differentiation, survival of various cell types^1–4^, and in several plasticity-related processes in the nervous system^5^. U0126 (1,4-diamino-2,3-dicyano-1,4-bis (2-aminophenylthio) butadiene) is widely used as a potent and selective non-competitive inhibitor of MEK^6^, therefore, the activation of its downstream target, MAPK/ERK. U0126 has been a valuable pharmacological tool for studying the ERK signaling pathway. The concentration of U0126 used to block the MAPK/ERK signaling pathway is typically 10 μM in cultured neurons^7,8^, and 20 μM in brain slices^9–11^. It has been demonstrated in various cell types that the ERK signaling pathway plays important roles in modulating K_V_^12–14^, synaptic plasticity^10,15–20^, and learning and memory^21–23^ (reviewed by^19,24,25^). On the other hand, U0126 is found to accelerate K_V_ inactivation in heterologous cells expressing several types of K_V_^26^. Therefore, it is of interest and importance to determine whether U0126 at a concentration thought to specifically inhibit MEK-MAPK signaling can have a significant effect on K_V_ in primary neuronal cultures and brain slices.

Voltage-gated potassium channels (K_V_) are key regulator of membrane excitability. Mammalian neurons express various types of K_V_ that exhibit different voltage- and time-dependent channels kinetics. K_V_ are multimeric proteins assembled from pore-forming α subunits and auxiliary β subunits. The α subunits of K^+^ channels are encoded by 12 subfamilies of genes (K_V_1–12)^27^. Previous studies have shown that the CA1 pyramidal neurons^28,29^, like many other types of neurons found in various brain regions^30,31^, express at least three major types of K_V_ currents; the transient fast-inactivating K^+^ current (I_A_), the delayed rectifier K^+^ current (I_DR_), composed of a non-inactivating, fast delayed rectifier K^+^ current (I_D_), and the slowly inactivating delayed rectifier K^+^ current (I_K_)^31^. K^+^ channels underlying those currents possess distinct biophysical properties, pharmacology, and molecular identity^32^. The I_A_, mainly assembled from K_V_4.2 and K_V_4.3^33^ of K_V_4 subfamily, are blocked by 4-AP but insensitive to TEA. I_A_ is rapidly activated upon depolarization and quickly recovers from inactivation, and therefore can influence action potential onset time, threshold, and inter-spike intervals as well as dendritic backpropagation action potentials^34^. I_D_, likely composed of K_V_3.1 and K_V_3.2 channels, exhibit rapid activation and deactivation and are highly sensitive to both TEA and 4-AP. It plays a prominent role in promoting high firing frequency^35^ and is highly enriched in fast-spiking inhibitory interneurons^36^. I_K_ presumably encoded by K_V_2 channels^29^, show intermediate TEA-sensitivity^37^ and slow activation time course. The K_V_2.1 are the predominant delayed rectifier K_V_ that regulate neuronal excitability, action potential duration, and tonic spiking^38^.

Here, we show that bath application of U0126 resulted in a dose-dependent inhibition of both the I_A_ and I_DR_ on primary hippocampal cultures and acute brain slices. This inhibiting effect of U0126 appeared to be of much higher potency (100- to 1000-fold) on the I_A_ and I_DR_ than the classical K_V_ blockers 4-AP or TEA.

## Results

### Dose-dependent blockade of K^+^ currents by U0126 in primary hippocampal neurons

**Figure 1:**
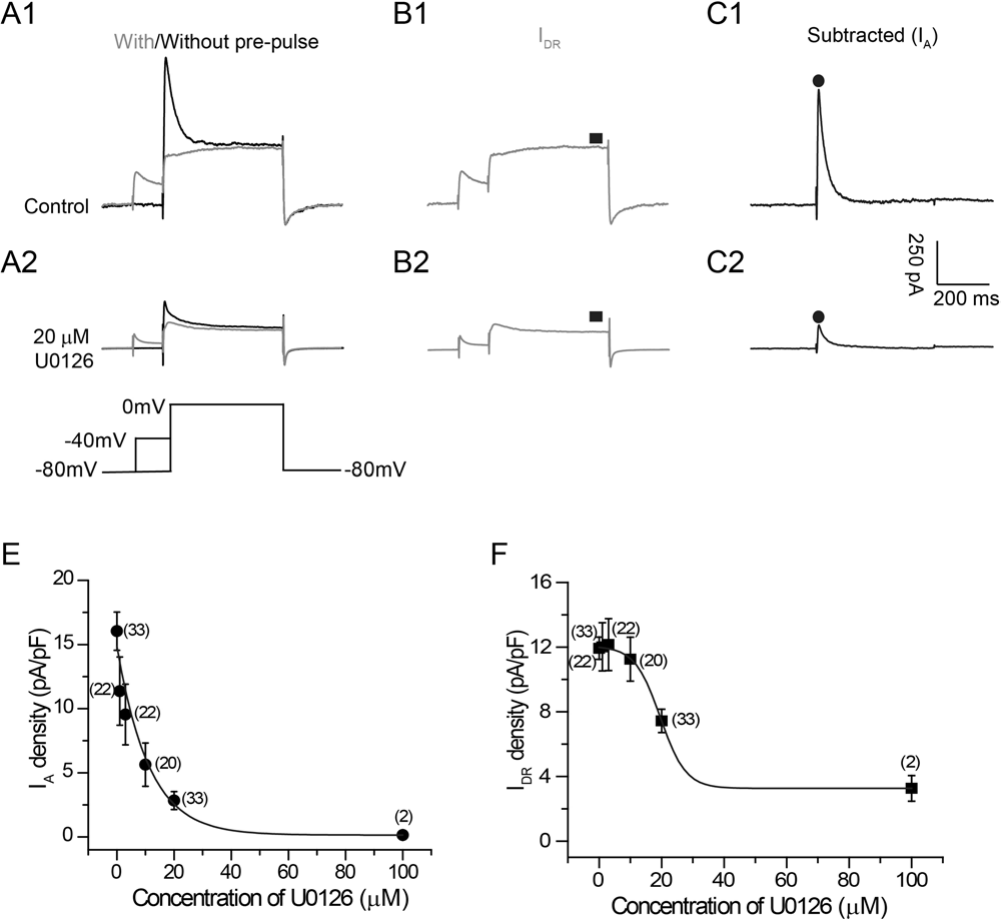
Non-selective block of voltage-dependent whole-cell K^+^ currents by U0126 in primary hippocampal neurons. (**A1**,**A2**) Representative traces showing superimposed currents with and without prepulse from control and U0126-treated neurons, respectively. Schematic diagram of the prepulse voltage protocol used to isolate I_A_ (bottom) is shown at the bottom of the traces. (**B1**,**B2**) Representative traces of I_DR_ in control and U0126-treated neurons, respectively. (**C1**,**C2**) Representative traces of I_A_ isolated from the voltage protocol under the control and U0126 treatment condition. The I_A_ were obtained by subtracting I_DR_ from the whole-cell K^+^ currents. I_A_ was measured at peak amplitude (indicated by a filled circle) and I_DR_ was measured by a small, late window as indicated by a filled square. (**D**,**E**) Dose–response curves of I_A_ and I_DR_ in the presence of U0126. The IC_50_ for I_A_ and I_DR_ were 9.5 ± 0.1 μM and 19.3 ± 0.4 μM, respectively.

**Figure 2:**
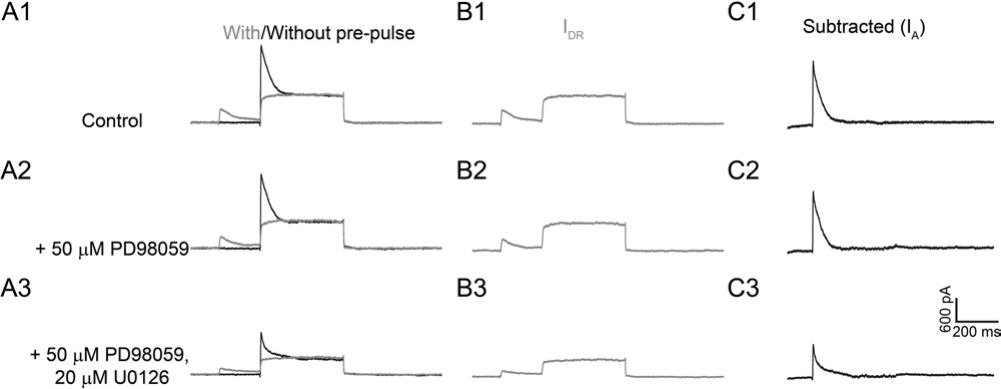
Lack of PD98059 effects on K^+^ currents. (**A1**–**C1**) Representative traces of the total K^+^ currents, I_DR_, and I_A_ under the control condition. (**A2**–**C2**) Application of 50 μM PD98059 produced no changes in I_A_ and I_DR_. (**A3**–**C3**) Subsequent addition of 20 μM U0126 to the same neuron resulted in dramatic reduction in peak amplitude of both I_A_ and I_DR_. Similar results were obtained in three additional neurons.

We first used primary culture of hippocampal neurons prepared from postnatal day 0–3 rats to test U0126 effects on K^+^ currents (Fig. 1). In voltage-clamp mode, two kinetically distinct K^+^ current components, the transient fast-inactivating K^+^ current, I_A_ and sustained, delayed rectifier K^+^ current, I_DR_ were identified^39^. A prepulse voltage protocol was used to isolate the I_A_ and I_DR_ (Fig. 1A1,A2)^40^. To selectively activate the I_DR_ (Fig. 1B1,B2), neurons were held at −80 mV and voltage was stepped to 0 mV with a prepulse to −40 mV in order to inactivate the I_A_. Subtracting I_DR_ from the total K^+^ current elicited by a voltage step yielded the I_A_ (Fig. 1C1,C2). Examining the dose-response relationship of I_A_ and I_DR_ showed a half maximal inhibitory concentration (IC_50_) at 9.5 ± 0.1 μM (Fig. 1D) and 19.3 + 0.4 μM (Fig. 1E), respectively. At 10 μM, a concentration commonly used in neuronal culture studies, U0126 produced a significant inhibition of the I_A_ (65 ± 11%, n = 20; p < 0.05) (Fig. 1D) but had no significant inhibition of the I_DR_ (6 ± 3%, n = 20; p > 0.05) (Fig. 1E). At 20 μM, U0126 significantly reduced both the I_A_ (82 ± 4%, n = 33; p < 0.05) (Fig. 1D) and I_DR_ (38 ± 6%, n = 33; p < 0.05) (Fig. 1E). Therefore, both I_A_ and I_DR_ can be inhibited by U0126, with the I_A_ being more sensitive to the inhibition of U0126. In contrast, PD98059, a structurally-unrelated MEK inhibitor, had no effects on K^+^ currents (Fig. 2). These results raise a possibility that U0126 could directly affect K_V_ at the concentration that is commonly used as a specific MEK inhibitor.

### U0126 blocked K^+^ channels with greater potency than 4-AP and TEA

**Figure 3:**
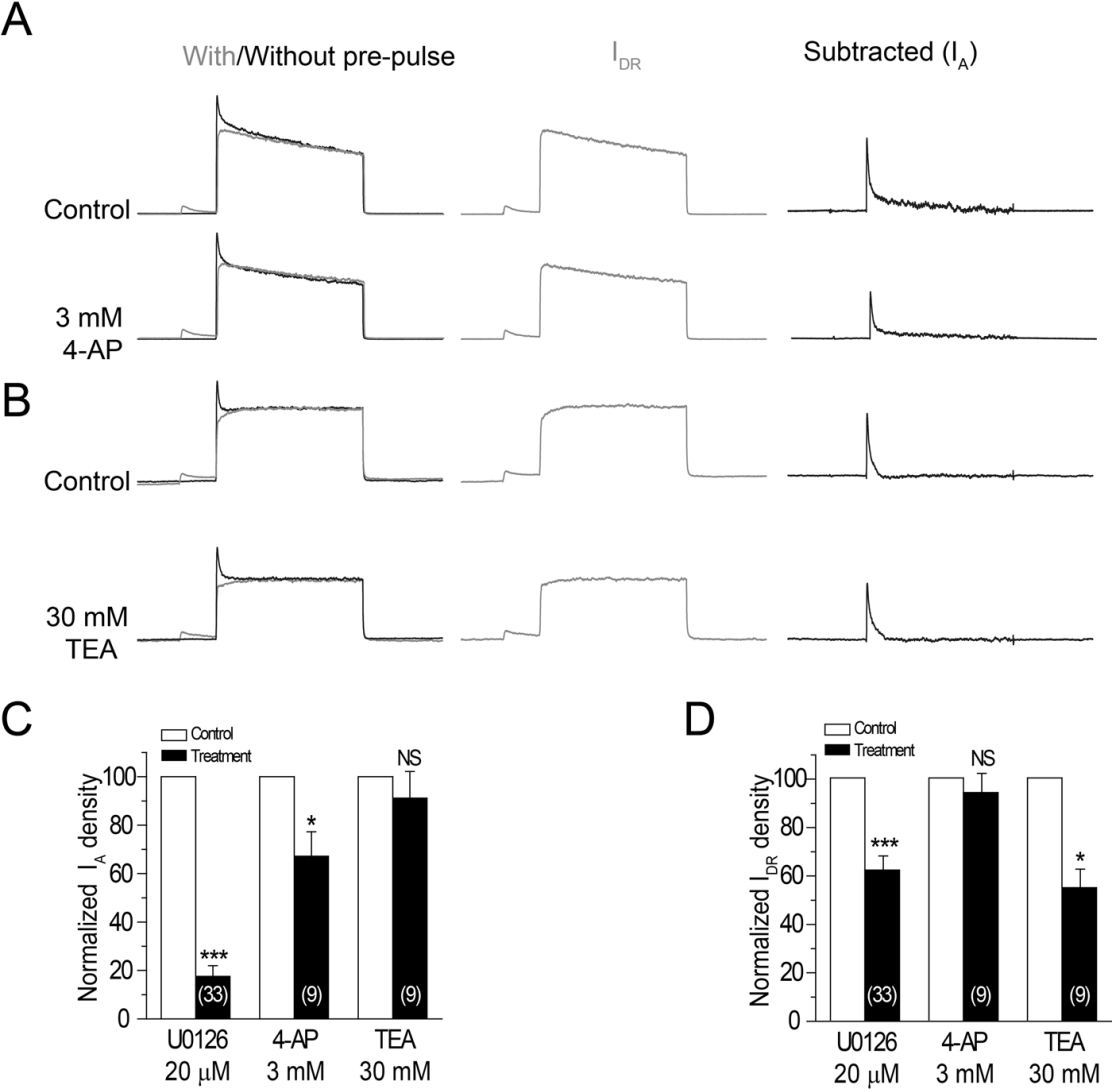
Comparison of inhibiting effects of U0126, 4-AP and TEA on I_A_ and I_DR_. (**A**) Representative K^+^ current traces recorded from a neuron before and after 4-AP application. (**B**) Representative K^+^ current traces recorded from a neuron before and after TEA application. Quantitative histograms of I_A_ (**C**) and I_DR_ (**D**) were constructed by normalizing current densities to that of control condition, i.e. before the application of drugs. Data are expressed as mean ± s.e.m. To determine statistical significance. One way-ANOVA was used, followed by the post hoc Scheffe’s test, *P < 0.05, ***P < 0.001.

We then compared the effects of U0126 on I_A_ and I_DR_ with classic K_V_ inhibitors, 4-AP and TEA. As shown in Fig. 3, 3 mM 4-AP produced 33 ± 10% (n = 9, p < 0.05) inhibition of the I_A_ (Fig. 3A) and 6 + 7% (n = 9, p > 0.05) inhibition of the I_DR_ (Fig. 3B); whereas 30 mM TEA produced inhibition of the I_DR_ 45 ± 8% (n = 9, p < 0.05) (Fig. 3B) and did not significantly change I_A_ (Fig. 3A). These results are consistent with well-established observations that 4-AP preferentially blocks I_A_^41^ and low levels of TEA have no effect on I_A_^42^ but partially inhibit the residual sustained current^43^. These results suggest that U0126 inhibits I_A_ and I_DR_ in the micromolar range of concentrations, exhibiting 100- to 1000-fold higher potency than the classical K_V_ blockers 4-AP and TEA, which work in millimolar range of concentrations.

### Prolongation of action potentials by U0126

**Figure 4:**
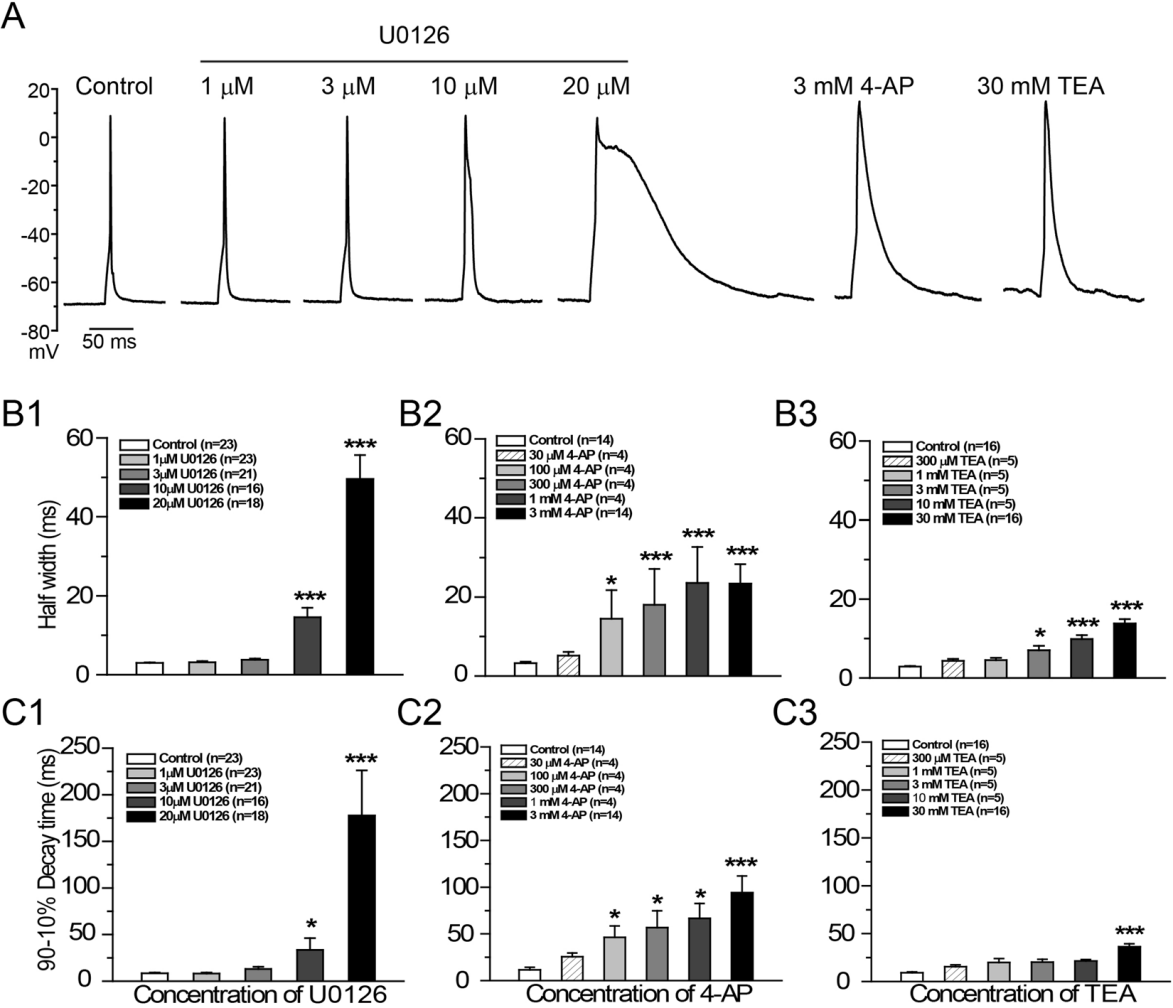
U0126 increases the decay time and half-width of evoked action potentials in primary hippocampal neurons. (**A**) Under current clamp, single action potentials were evoked by injecting a 20-ms depolarizing currents at 10 pA steps from the resting membrane potential. Representatives of typical traces of control, during the application of 1 μM U0126, 3 μM U0126, 10 μM U0126, 20 μM U0126, 3 mM 4-AP and 30 mM TEA were shown. These traces were all obtained from the same cell. (**B1**–**B3**) Show quantification of the half-width of action potentials during application of various concentrations of U0126, 4-AP and TEA, respectively. (**C1**–**C3**) Show the quantification of the decay time of action potentials during application of various concentrations of U0126, 4-AP and TEA, respectively. P indicates statistical significance using One way-ANOVA followed by the post hoc Scheffe’s test, *P < 0.05 vs. control group, ***P < 0.001 vs. control group.

We next studied the effects of U0126 on action potentials under whole-cell current-clamp recording mode. To elicit action potentials, a series of 20 ms depolarizing current pulses in 10 pA steps from resting membrane potential were injected into cultured hippocampal neurons. To inhibit synaptic activity, 20 µM CNQX (an AMPAR antagonist) and 100 μM APV (a potent and selective antagonist for NMDA receptors) were added to the bath. Consistent with its inhibitory effects on K^+^ currents, bath perfusion of U0126 increased the half-width and decay time of action potential in a dose-dependent manner (Fig. 4A). With exposure to 1 and 3 μM U0126, half-width and decay time of action potentials showed no significant difference to that of controls. At 10 μM, action potential half-width was increased by 392 ± 82% (n = 16, p < 0.001) and decay time increased by 298 ± 153% (n = 16, p < 0.05); At 20 μM, U0126 had a dramatic effect, increasing the action potential half-width by 1573 ± 205% (n = 18, p < 0.001) and decay time by 2023 ± 573% (n = 18, p < 0.001) (Fig. 4B1,C1). In a comparison, 3 mM 4-AP or 30 mM TEA increased the action potential decay time by 710 ± 155% (n = 14, p < 0.001) or 296 ± 35% (n = 16, p < 0.001), respectively. Below 10 mM, TEA had no significant effect on decay time of action potentials (Fig. 4C3). These results are consistent with several previously reported effects of 4-AP and TEA on action potentials^42,44^. Therefore, we conclude that U0126 prolongs the action potential half-width and decay time much more efficiently than 4-AP and TEA, possibly through its high potency on K^+^ current inhibition.

### Suppression of action potential firing frequency by U0126

**Figure 5:**
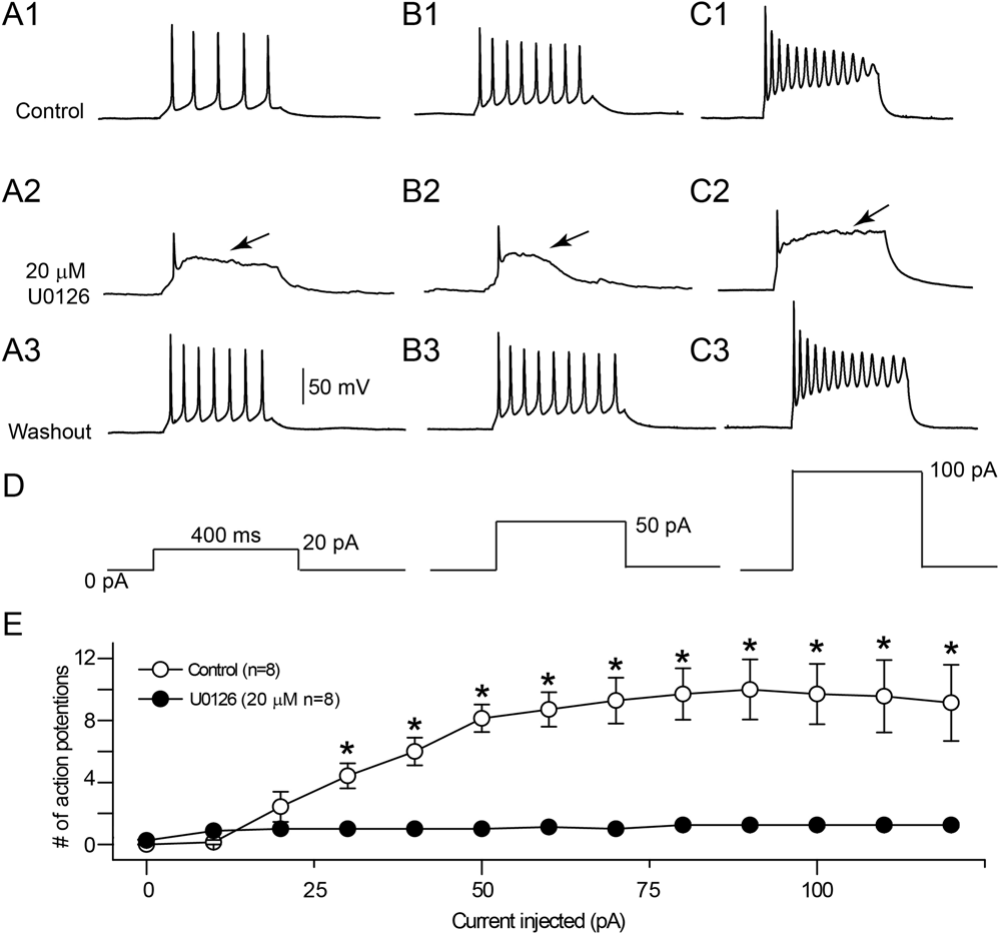
Effects of U0126 on action potential waveforms and firing patterns in primary hippocampal neurons. (**A**–**C**) Representative traces of whole-cell currents from control neurons (**A**), neurons treated by U0126 (20 μM) (**B**) and washout of U0126 (**C**). The voltage changes of cultured hippocampal neurons in response to a series of a 400 ms-duration depolarizing current pulse (0 pA to +120 pA in 10 pA steps) injected into neurons at rest membrane potential (RMP) were recorded and shown at 20 pA (left); 50 pA (middle) and 100 pA (right) levels. (**D**) shows quantitative plots of the current versus spike firing frequency relationships (**I**–**F**) curves. The number of action potentials elicited were counted and plotted against the levels of injected currents. Notice that after bath application of 20 μM U0126, only a single action potential followed by a prolonged membrane depolarization could be evoked no matter how much depolarizing currents were injected. Bars indicate standard error of the mean; N = 8 for all conditions. P indicates statistical significance using Two way-ANOVA followed by the post hoc Scheffe’s test, *P < 0.05.

We further examined the potential effect of U0126 on the action potential firing pattern by analyzing the current-frequency (I-F) relationship. Under control conditions, the number of action potentials increased progressively with the increase of current injection steps over the course of 400 ms depolarizations from 0 to + 120 pA (Fig. 5A1,B1,C1 and E). In contrast, in the presence of 20 μM U0126, regardless the strength of current injections, only a single action potential was evoked, followed by a prolonged membrane depolarization (Fig. 5A2,B2 and C2). After washout of U0126 for 5–10 minutes, the repetitive firing pattern of action potentials was restored, similar to that of the control condition (Fig. 5A3,B3 and C3). We conclude that U0126 exerts profound influence on membrane excitability and firing pattern of cultured hippocampal neurons.

### Suppression of K^+^ currents by U0126 in acute hippocampal slices

**Figure 6:**
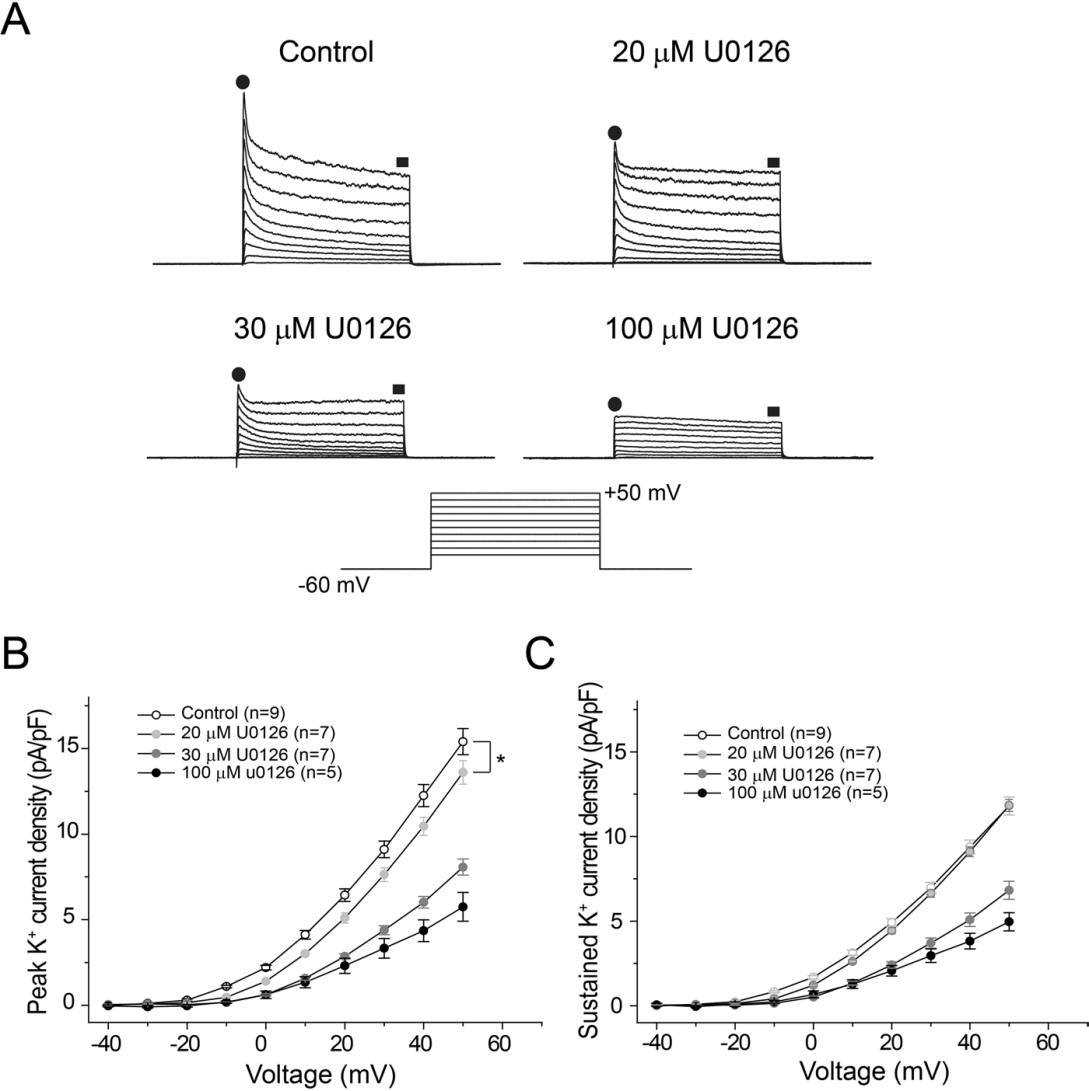
Suppression of K^+^ currents by U0126 in acute hippocampal slices. (**A**) Representative traces of whole-cell voltage-gated K^+^ currents recorded from control pyramidal neurons, neurons treated by 20 μM U0126, 30 μM U0126 and100 μM U0126, as indicated. The schematic diagram of the voltage protocol used to evoke K^+^ currents is shown at the bottom of the traces. We recorded K^+^ currents in CA1 neurons in response to a voltage step from −40 mV to +50 mV with 10 mV increments. (**B**) Quantitative comparison of the current density of early K^+^ currents (measured at peak amplitude, indicated by filled circles) in CA1 neurons at different conditions as indicated. (**C**) Quantitative comparison of the current density of the late, sustained K^+^ currents (measured indicated by filled square) in CA1 neurons at different concentrations after bath application of U0126. P indicates statistical significance using Two way-ANOVA followed by the post hoc Scheffe’s test, *P < 0.05.

Based on the above results, we further examined the effects of bath perfusion of U0126 on K^+^ currents in CA1 pyramidal neurons of acute hippocampal slices. Both I_A_ and I_DR_ are highly expressed in CA1 pyramidal neurons and are important determinants of their membrane excitability^45^. Consistent with the effects of U0126 on K^+^ currents in primary hippocampal cultures, U0126 similarly inhibited the K^+^ currents of CA1 pyramidal neurons in a dose-dependent manner in acute slices. As shown in Fig. 6, bath application of 20 μM U0126 produced a small but significant reduction of the current density of the early K^+^ currents (measured at peak of the currents, presumably largely composed by I_A_: 15.4 ± 0.76 pA/pF in control; n = 9, at + 50 mV vs 13.6 ± 0.68 pA/pF in U0126; n = 7, p < 0.05), whereas the late sustained currents, presumably the I_DR_, were not affected (Fig. 6B). At 30 μM and 100 μM, U0126 further reduced the current density of the early K^+^ currents by 47.6 ± 3% and 62.7 ± 5% (at +50 mV), respectively; meanwhile, the late, sustained K^+^ currents (measured indicated by filled squares, Fig. 6A) showed significant reduction compared to control (Fig. 6C). These results demonstrate that U0126 also non-selectively blocks native K^+^ currents in brain slices.

### Effects of U0126 on action potential waveform and firing patterns in hippocampal neurons

**Figure 7:**
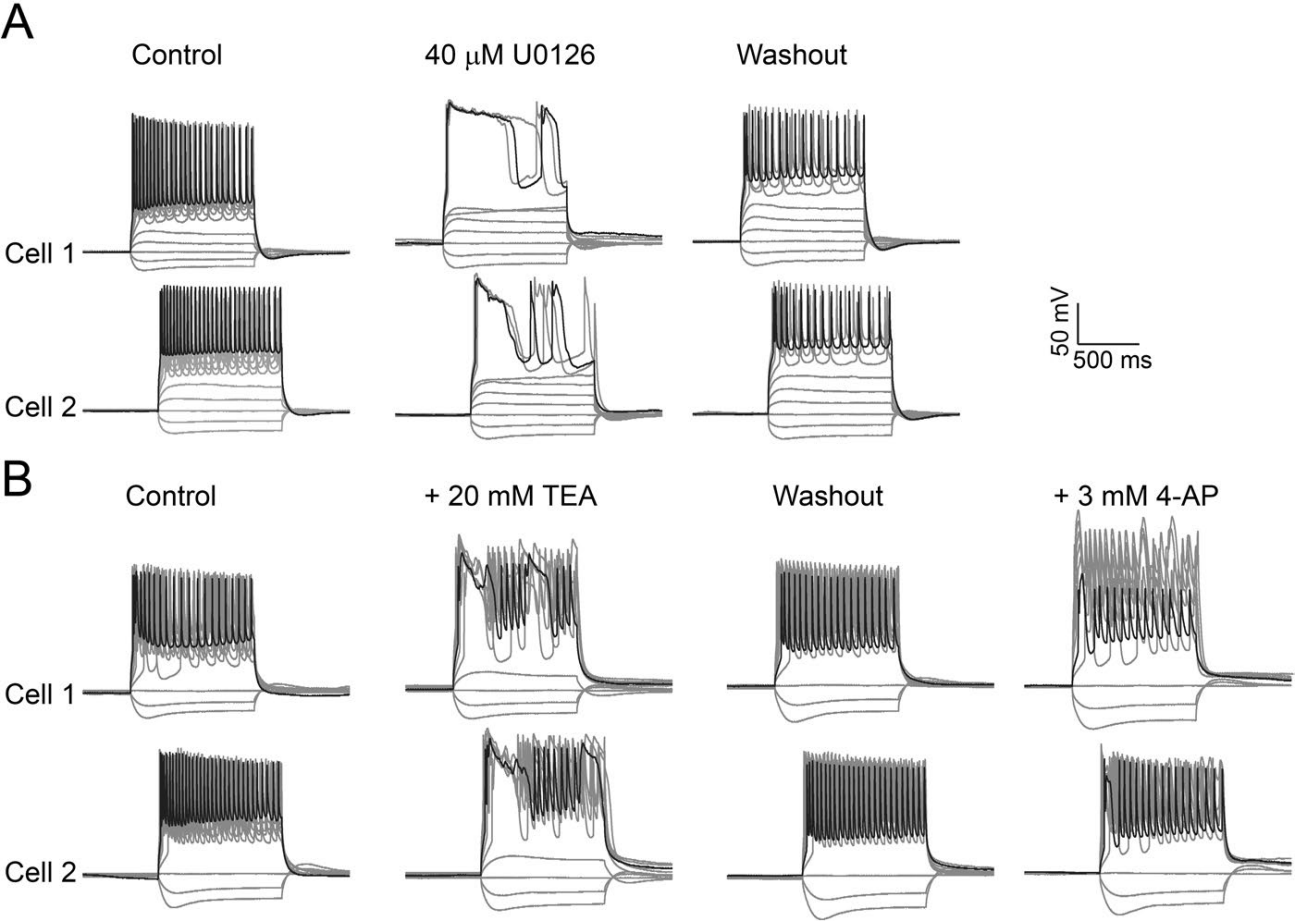
Effects of U0126 on action potential waveforms and firing patterns in hippocampal neurons. (**A**) Representative double whole-cell current clamp recordings from two regular-spiking pyramidal neurons from control, neurons treated by 40 μM U0126 and washout, as indicated. These tracings were obtained from the same neuron. (**B**) Representative double whole-cell current clamp recordings from two regular-spiking pyramidal neurons from control, neurons treated by 20 mM TEA, washout and treated by 3 mM 4-AP, as indicated. These tracings were obtained from the same neuron. Recordings were made from P20 mouse hippocampus. The current step is 20 pA (from −40 pA to 140 pA).

Lastly, we examined the functional consequence of U0126 blockade of K^+^ channels, specifically on action potential (AP) waveform and firing patterns of hippocampal neurons in acute brain slices. To this end, we recorded two regular-spiking pyramidal neurons simultaneously from P20 mouse slices. Under control conditions, those neurons exhibit a regular firing pattern in response to depolarizing current pulses of 1 s. Bath application of 40 μM U0126 dramatically reduced the maximal firing rate from about 18–20 Hz to roughly 2–3 Hz in response to current injections of the same amplitude. Action potential waveform was also greatly broaden with a prolonged half-width and a slower decay time. Washout of U0126 for 30 minutes restored AP waveform and firing patterns to the control condition (Fig. 7A). We then compared the effects of U0126 on AP waveform and firing patterns with classic K_V_ inhibitors, 4-AP and TEA. As shown in Fig. 7B, after adding 20 mM TEA, the two pyramidal neurons reduced their maximal firing rate from ~18–20 Hz in control to 8–9 Hz in response to current injections. After washout of TEA for 30 minutes, AP waveform and firing patterns were returned to the control condition. Subsequent addition of 3 mM 4-AP reduced the maximal firing rate reduced to 14–16 HZ. Taken together, these results suggest that U0126 is more efficacious than 4-AP and TEA in suppressing maximal firing rate of pyramidal neurons in hippocampal slices.

## Discussion

Our major finding is that U0126, a widely-used MEK inhibitor acts to serve as a highly potent non-selective K_V_ blocker. Consequently, it changes neuronal excitability and firing pattern at the concentrations commonly used in many previous studies. Our results thus raise cautions for using U0126 as specific inhibitor for studying MEK-MAPK signaling in neurons.

We demonstrated that bath perfusion of U0126 significantly inhibits the I_A_ and I_DR_ in hippocampal neurons in a dose-dependent manner in primary neuronal cultures as well as acute brain slices. Remarkably, U0126 (in micromolar range of concentrations) inhibits both the I_A_ and I_DR_ with much higher potency (100- to 1000-fold) than the classical K_V_ blockers 4-AP and TEA, which preferentially inhibits the I_A_ and I_DR_ in the millimolar range of concentrations, respectively. Consistently, U0126 showed clear effects on action potential waveform and the ability of firing repetitive action potentials. Bath perfusion of U0126 not only increased the half-width and decay time of individual action potentials in a dose-dependent manner (Fig. 4), but also dramatically reduced firing frequency in response to long current pulse injections (Figs 5 and 7A). Interestingly, U0126’s reversible channel inhibiting effect was not mimicked by PD98059, a structurally-unrelated MEK inhibitor (Fig. 2), indicating the effect of U0126 was independent of its inhibition on MEK.

In our study, we did not differentiate I_DR_ into I_D_, I_K_ components. It would be interesting to further evaluate if U0126 exhibits similar or different inhibitory effects on I_D_, I_K_. Currently, the mechanism of U0126 blocking K_V_ is not clear. In heterologous cells expressing the A-type K^+^ channels, K_V_4.2 and K_V_4.3 as well as K_V_1.1, a typical non-inactivating K_V_, U0126 exhibits its inhibition on K_V_ by accelerating the inactivation of these different channels^26^. The authors further demonstrated that the action of U0126 is likely a result from a combination of open channel blocking and modulation of channel-gating but independent on its inhibitory effect on ERK or the phosphorylation of the channels. Together with our current observation that U0126 exhibits non-selective inhibitory effects on I_A_ and I_DR_, we postulate that U0126 may be able to directly bind and block the channel pore of various native K_V_ or alter channel gating by interacting with membrane lipids^46^.

Several studies have reported that direct phosphorylation of K_V_4.2 potassium channel by ERK/MAPK can inhibit I_A_ in different subtypes of neurons including the hippocampal CA1 pyramidal neurons^13,47–49^. In particular, Watanabe^49^ showed that application of 20 μM U0126 to the outside of the neurons or included in the whole cell patch pipette produced a small but significant leftward shift in the activation voltage and an overall reduction in I_A_. In our study, we found that 20 μM U0126 produced a small but significant reduction of the current density of peak K^+^ currents (Fig. 6B). At higher concentrations, U0126 exhibited a greater inhibition on the late, sustained K^+^ currents in a concentration-dependent manner (Fig. 6C). There is no clear explanation for this discrepancy. Differences in species, age (5–8 week-old mature rats used by Watanabe *et al*.^49^ vs 2–3 week-old young mice used in our study), methodology such as perfusion speed, internal solution or different subtypes of pyramidal neurons recorded, are all plausible factors for the discrepancy.

K_V_ blockers have been considered as potential drugs for many diseases (Tian *et al*., 2014) associated with low excitability or compromised motor functions such as multiple sclerosis^50^, spinal cord injury^51^ or Parkinson’s Disease^52^. For example, 4-AP has been used clinically in treating Lambert-Eaton myasthenic syndrome and multiple sclerosis^53–55^; and TEA was the first “ganglionic blocker” drug to be introduced into clinical practice^56^ but their uses have been limited due to toxicity^57,58^, and were soon replaced by other drugs. Because of significant adverse side effects and a narrow range for a safe clinical dose, 4-AP failed in clinical trials in treating patients with spinal cord injuries^59,60^. As discussed above, 4-AP or TEA typically acts on K_V_ in the millimolar range of concentrations (Fig. 3), whereas U0126 inhibits both the transient A-type and the residual sustained K^+^ currents in the micromolar range of concentrations as shown in this study (Fig. 1). Such a high potency (100- to 1000-fold) and broad-spectrum action may prove to be favorable properties as a potential new class of K_V_ blockers in treating those aforementioned diseases. Indeed, our ongoing study indicates that, beside a powerful enhancement on neuronal excitability, U0126 also greatly potentiates presynaptic release, hence evoking synchronized firing in immature cultured hippocampal neurons (unpublished data). We believe that a further understanding of the structural basis and blocking mechanisms of U0126 and its analogs as potent, non-selective inhibitors of K_V_ and the removal of its unwanted action on MEK should not only provide useful insights into the structure-function of K_V_ in general, but also may prove valuable in developing more efficacious and safe new K_V_ blockers in treating various relevant diseases due to conduction deficits and low excitability in the excitable organs including brain, heart and muscle.

In conclusion, we have observed dramatic effects of U0126 on K_V_ in primary hippocampal cultures and brain slices. The data reported here suggest that cautions should be taken when interpreting experimental results using U0126. Furthermore, our finding that U0126 exhibits much higher potency on K_V_ than 4-AP and TEA raises the possibility that U0126 and its derivatives can be further developed as a potent, new class of K_V_ blockers and may provide a new perspective in the development new treatments for various neuromuscular and neurodegenerative diseases.

## Methods

### Hippocampal primary culture

All animals were housed and maintained in accordance with procedures approved by the Ethics Committee for animal research at South China Normal University, in line with the Guidelines for Animal Care established by the National Institute of Health. All experimental procedures were approved by the Ethics Committee for animal research at South China Normal University. Hippocampal cell cultures were prepared as described previously^8^. Briefly, hippocampal CA1/CA3 regions were dissected from 0- to 3-days-old C57BL6/J mouse, dissociated by trypsin XI treatment followed by trituration with a siliconized Pasteur pipette, and then plated onto coverslips coated with Matrigel (Bd Biosciences, USA). Culture medium consisted of minimal essential medium (Invitrogen, CA), 0.6% glucose, 0.1 mg/l bovine transferrin (Calbiochem, CA), 0.25 mg/l insulin (Sigma-Aldrich, USA), 0.3 mg/l glutamine, 5–10% fetal bovine serum (Sigma-Aldrich, USA), 2% B-27 supplement (Invitrogen, USA), and 2 μM cytosine β-D-arabinofuranoside (Sigma-Aldrich, USA). Cultures were maintained at 37 °C in a 95% air, 5% CO_2_-humidified incubator. Cultures of 7–9 DIV (days *in vitro*) were used for whole-cell patch clamp recordings.

### Patch-clamp recording

#### Whole-cell patch-clamp recording in hippocampal cultures

Traditional whole-cell patch-clamp recordings were performed from cultured hippocampal neurons plated on coverslips, which were placed in a recording chamber mounted on a fixed-stage inverted phase-contrast microscope (Nikon, Japan). Patch electrodes (3–5 MΩ) were made from borosilicate glass (WPI, USA). Whole-cell capacitance and series resistances were recorded and compensated to >80%, and in addition, series resistances were less than two times the tip resistance. The Tyrode’s bath solution contained (in mM): 129 NaCl, 5 KCl, 2 CaCl_2_, 1 MgCl_2_, 0.01 glycine, 30 D-glucose and 25 HEPES, pH 7.2–7.4. The pipette solution contained (in mM): 110 K-gluconate, 40 HEPES, 10 EGTA, 2 Na_2_-ATP, 2 Mg-ATP and 0.3 GTP, pH 7.35 (adjusted with KOH). To isolate K^+^ currents in pyramidal neurons, 1 µM tetrodotoxin (TTX) and 2 mM MnCl_2_ were added to the bath to block Na^+^ currents, Ca^2+^ currents, and Ca^2+^-activated K^+^ currents^40^. To isolate the fast inactivating transient current (I_A_), cells were held at −80 mV, and the voltage was stepped to 0 mV with or without a prepulse to −40 mV^40^. The difference between the currents elicited with and without the prepulse was measured as I_A_. Leakage and capacitive currents were digitally subtracted on-line with P4 protocol^40^.

#### Whole-cell patch-clamp recording in acute hippocampal slices

Coronal brain slices (350 μm thick) were prepared from 15- to 21-days-old C57BL6/J mice in cutting solution with a vibratome (Lecia VT1000S, Germany). The cutting solution contained (in mM): 210 Sucrose, 26 NaHCO_3_, 3 MgSO_4_·7H_2_O, 0.75 CaCl_2_, 1 NaH_2_PO_4_·2H_2_O, 3 KCl and 10 D-glucose. Slices were incubated at room temperature for 1 hour in the artificial cerebrospinal fluid (ACSF). The ACSF contained (in mM): 124 NaCl, 2.5 KCl, 1.25 NaH_2_PO_4_·2H_2_O, 1.3 MgSO_4_·7H_2_O, 26 NaHCO_3_, 10 D-glucose and 2.5 CaCl_2_. The pipette solution contained (in mM): 110 K-gluconate, 40 HEPES, 10 EGTA, 2 Na_2_-ATP, 2 Mg-ATP and 0.3 GTP, pH 7.35 (adjusted with KOH). At a holding potential of −60 mV, the K^+^ currents were evoked by voltage steps (from −40 mV to +50 mV in 10 mV increments, 400 ms) in the presence of TTX (1 μM) and CdCl_2_ (2 mM) to block voltage-activated Na^+^ and Ca^2+^ currents, as well as Ca^2+^-activated K^+^ currents^61^.

### Drugs

U0126 (Sigma-Aldrich, USA and Calbiochem, CA), PD98059 (Calbiochem, CA), 4-AP and TEA (Sigma-Aldrich, USA) were dispersed in ultrapure water or DMSO as stock solution. Stock solutions were dissolved directly in the Tyrode or ACSF at the desired concentration. Neurons were exposed to different drug solutions until the steady-state effects were obtained, using a perfusion system.

### Data analysis

Patch-clamp data were processed by using Clampfit 10.2 (Molecular Devices, USA) and then analyzed in Origin 8 (OriginLab, USA). Dose–response curves were fitted by non-linear curve fitting of the Boltzmann equation to the data using the Origin software. The values were presented as means ± SE. Analysis of variance (ANOVA) followed by the post hoc Scheffe’s test was used for statistical analysis. Changes were considered significant when P < 0.05.
